# Old zoonotic agents and novel variants of tick-borne microorganisms from Benguela (Angola), July 2017

**DOI:** 10.1186/s13071-022-05238-2

**Published:** 2022-04-21

**Authors:** Ana M. Palomar, Israel Molina, Cristina Bocanegra, Aránzazu Portillo, Fernando Salvador, Milagros Moreno, José A. Oteo

**Affiliations:** 1 Infectious Diseases Department, Center of Rickettsiosis and Arthropod-Borne Diseases (CRETAV), San Pedro University Hospital-Center of Biomedical Research From La Rioja (CIBIR), Piqueras, 98, 26006 Logroño, La Rioja Spain; 2 Infectious Diseases Department, Vall d’Hebron University Hospital, PROSICS Barcelona, 08035 Barcelona, Spain; 3 Hospital Nossa Senhora da Paz, Cubal, Angola

**Keywords:** Ticks, Tick-borne microorganisms, Zoonotic agents, *Rickettsia*, Anaplasmataceae, *Coxiella*, *Borrelia*, *Spiroplasma*, *Babesia*, Angola

## Abstract

**Background:**

Ticks and tick-borne diseases constitute a real threat for the livestock industry, which is increasing in Angola. In addition, ticks are vectors of zoonoses of public health concern, and scarce information is available from this country. In an effort to contribute to the prevention of zoonotic infectious diseases affecting humans and animals, the molecular screening of certain tick-related microorganisms collected on cattle in Angola was performed under a ‘One Health’ scope.

**Methods:**

Ticks collected from cattle in Cubal (Benguela Province, Angola) in July 2017 were analysed in pools using specific PCR assays for bacteria (*Rickettsia*, Anaplasmataceae, *Borrelia*, *Coxiella* and *Spiroplasma*) and protozoa (*Theileria* and *Babesia*) detection.

**Results:**

A total of 124 tick specimens were grouped in 25 pools (two *Amblyomma variegatum*, three *Hyalomma truncatum*, 16 *Rhipicephalus decoloratus*, two *Rhipicephalus duttoni*, one *Rhipicephalus evertsi mimeticus* and one *Rhipicephalus* sp.). The amplified microorganisms were (pools): *Rickettsia africae* (two *A. variegatum* and one *R. decoloratus*), *Rickettsia aeschlimannii* (three *H. truncatum*), *Ehrlichia* spp. (six *R. decoloratus*), *Coxiella* spp. (all but *H. truncatum*), *Francisella* sp. (one *H. truncatum*), *Spiroplasma* sp. closely related to *Spiroplasma ixodetis* (three *R. decoloratus*), *Babesia bigemina* (two *R. decoloratus*) and *Babesia* spp. (two *A. variegatum*). The obtained nucleotide sequences from *Ehrlichia* spp., two *Coxiella* genotypes (from *R. duttoni* and *Rhipicephalus* sp.), *Francisella* sp. and *Babesia* spp. (from *A. variegatum*) reached low identities with known genetically characterized species.

**Conclusions:**

This study demonstrates the circulation in Angola of the pathogen *R. aeschlimannii* and potential novel tick-related microorganisms belonging to *Ehrlichia*, *Coxiella*, *Francisella*, *Spiroplasma* and *Babesia* spp. and corroborates the presence of *R. africae* and *B. bigemina*. Our results should be considered in developing protocols for the management of fever of unknown origin and for veterinary practices. Further studies are required to evaluate the risk of tick-borne diseases in Angola.

**Graphical Abstract:**

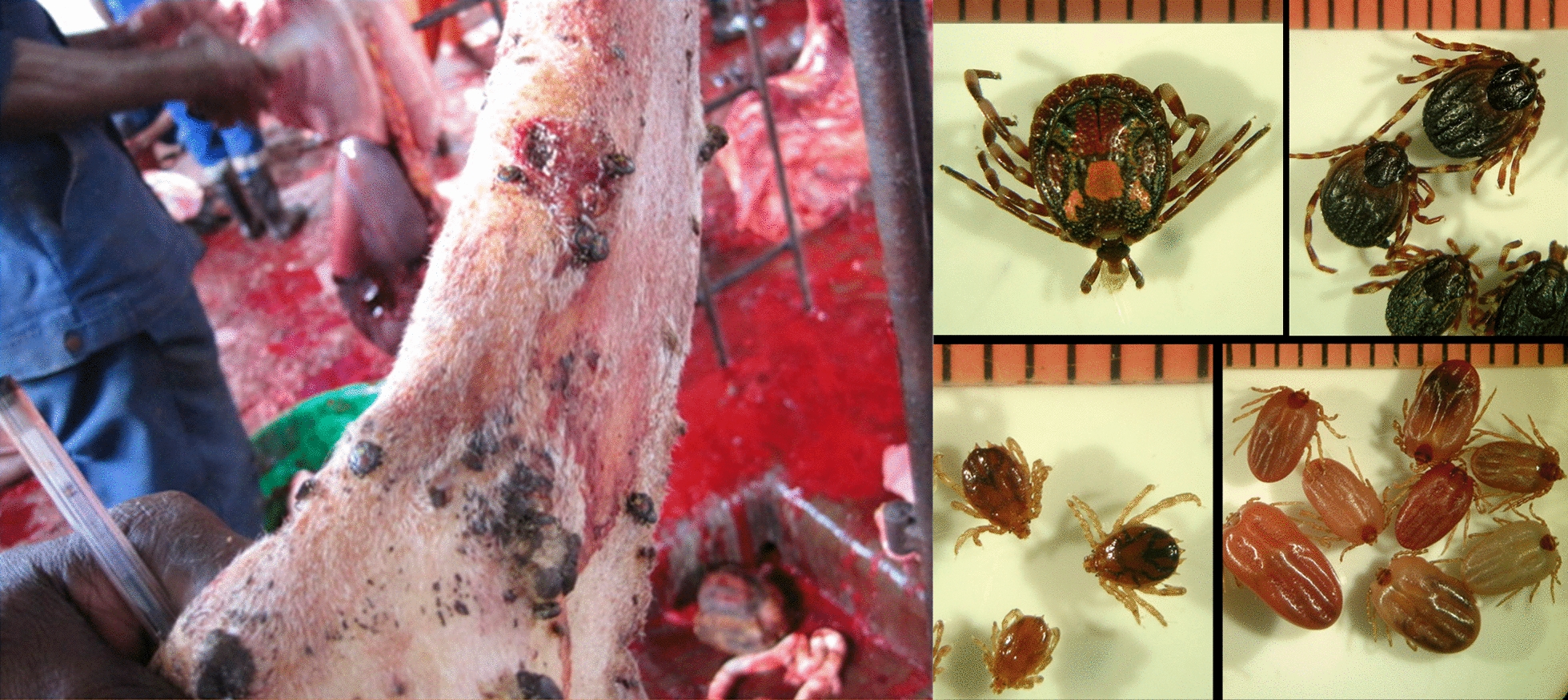

**Supplementary Information:**

The online version contains supplementary material available at 10.1186/s13071-022-05238-2.

## Background

Epidemics and pandemics have been present throughout history, but the ongoing COVID-19 pandemic and recent epidemics have strengthened the importance of ‘One Health’ to prevent spillover events [1]. Human/animal health and the environment are interconnected, and factors such as globalization, climate change, changes in land use and population growth could trigger new zoonotic outbreaks [2]. Early detection and knowledge of potential zoonotic agents, including vector-borne microorganisms, is crucial to implementing containment measures and preventing related infectious diseases. Ticks are prominent vectors of zoonoses that pose a public health risk, such as Crimean-Congo haemorrhagic fever that is considered a ‘priority disease’ by the World Health Organization, due to their epidemic potential and/or lack of sufficient countermeasures [3, 4]. Thus, surveillance systems for vectors and their microorganisms are critically needed.

Zoonotic agents, often underdiagnosed due to lack of diagnostic resources, are a known major cause of disease in sub-Saharan Africa, and studies have revealed the need for improved protocols for fever of unknown origin (FUO) management [5]. Tick-borne relapsing fever, rickettsiosis and babesiosis have been reported from southern Africa [5, 6], but tick-borne diseases from Angola are hardly known. Ticks are of unquestionable veterinarian concern worldwide and constitute a real threat for the livestock industry, with a higher impact in poor countries [7]. Diseases such as heartwater (caused by *Ehrlichia ruminantium*) or theileriosis (caused by *Theileria* spp.) are endemic in sub-Saharan Africa [7, 8]. The Angolan livestock population is increasing (https://www.fao.org/faostat/en/#data/QCL), mainly based on cattle production, and the expansion of livestock industry is linked to the incidence of zoonosis [9]. Therefore, the molecular screening of selected microorganisms of public health concern in ticks infesting cattle during a week of July 2017 in the slaughterhouse of Cubal (Angola) is reported.

## Methods

Ticks were collected from cattle in a slaughterhouse of Cubal (Benguela Province, Angola) from 1 to 8 July 2017, and preserved in 70% ethanol. Specimens were classified using a taxonomic key [10]. Selected individuals (at least two specimens from each morphologically classified species and those doubtful according to morphological features) were genetically characterized by polymerase chain reaction (PCR) of mitochondrial genes (Additional file 1: Table S1) using individual DNA. DNA was extracted from a leg of each specimen using two incubations of 20 min each with 100 µL of ammonium hydroxide 0.7 M at 100 and 90 °C, respectively [11]. Furthermore, tick halves were pooled (1–9 specimens) according to species and developmental stages. DNA from pools was extracted using a DNeasy Blood & Tissue kit (Qiagen), following the manufacturer’s recommendations with overnight lysis. Mitochondrial 16S rRNA PCRs were performed as controls of pool extractions (Additional file 1: Table S1). Bacteria (*Rickettsia*, Anaplasmataceae, *Borrelia*, *Coxiella* and *Spiroplasma*) and protozoa (*Theileria* and *Babesia*) were screened using specific PCR assays. Pan-bacterial 16S rRNA PCR was also performed (Additional file 1: Table S1).

The PCR amplicons obtained with the expected size were sequenced in forward and reverse senses. The nucleotide sequences were analysed using BioEdit v.7.2.6 software [12]. The consensus sequences produced were compared with those available in NCBI using BLAST [13], and submitted to GenBank when different. Clustal Omega [14] was used for multiple sequence alignment. Phylogenetic analyses were conducted with MEGA X [15] using maximum likelihood method including all sites. Confidence values for individual branches of the resulting trees were determined by bootstrap analysis (500 replicates).

## Results

A total of 124 ticks (five nymphs, 28 males and 91 females) were collected and morphologically classified as six *Amblyomma variegatum*, six *Hyalomma truncatum*, 107 *Rhipicephalus decoloratus* and five *Rhipicephalus* spp. Whenever performed, genetic characterization confirmed morphological identification, and also allowed the identification of three *Rhipicephalus duttoni* and one *Rhipicephalus evertsi mimeticus* (Tables 1, 2) among those *Rhipicephalus* spp.

**Table 1:** Comparison (% identity) of the studied Angolan tick mitochondrial amplicons with available GenBank sequences

Tick species	% Identity (bp)-GenBank accession no. (no. of analysed amplicons)		
	16S RNA	12S RNA	COI
*A. variegatum*	99.0 (404/408)-L34312 (3)	99.4 (339/341)-HQ856466 (3)	99.3 (560/564)-MK648415 (1)
*H. truncatum*	99.8 (401/403)-LC634545 (2)	100 (341/341)-AF150031 (2)	99.2–99.4 (617/622–670/674)-KY457529 (2)
*R. decoloratus*	99.5–99.8 (399–400/401)-KY457525 (4)	99.7 (343/344)-NC_052828 (4)	99.4–99.1 (616/620–652/658)-NC_052828 (3)
*R. evertsi mimeticus*	99.7 (370/371)-MF425975 (1)	100 (318/318)-AF031862 (1)	NA
*R. duttoni*	99.7 (352/353)-MW080164 (3)	98.7 (310/314)-MF425966 (1)	NA
*Rhipicephalus* sp.	97.0 (393/405)-LC634554^a^ (1)	98.2 (333/339)-KY457542^1^ (1)	NA

bp: base pairs; *A.*: *Amblyomma*; *H.*: *Hyalomma*; *R.*: *Rhipicephalus*; NA: not amplified

^a^*Rhipicephalus simus*

**Table 2:** Microorganisms amplified in this study

Microorganisms	Target gene	*Amblyomma variegatum* (2: 5N, 1M)^a^	*Hyalomma truncatum* (3: 1M, 5F)^a^	*Rhipicephalus decoloratus* (16: 24M, 83F)^a^	*Rhipicephalus duttoni* (2: 1M, 2F)^a^	*Rhipicephalus evertsi mimeticus* (1: 1F)^a^	*Rhipicephalus* sp. (1: 1 M)^a^
*Rickettsia* spp.	*ompA*	*R. africae* (100;CP001612) 2 (5N, 1M)	*R. aeschlimannii* (100;HQ335157) 3 (1M, 5F)	*R. africae* (100; CP001612) 1 (9M)	–	–	–
*Anaplasma*/*Neoehrlichia*/*Ehrlichia* spp.	*gro*ESL	–	–	*Ehrlichia* spp. (100; MW054557) 6 (44F)	–	–	–
*Ehrlichia* spp.	*gltA*	NP	NP	*Ehrlichia* spp. (96.9–97.0; KX987353)^b^ 6(44F)	NP	NP	NP
	16S rRNA^c^	NP	NP	*Ehrlichia* sp. (99.9; AF497581) 1 (9F)^d^	NP	NP	NP
*Borrelia* spp.	*flaB*	–	–	–	–	–	–
	*glpQ*	–	–	–	–	–	–
*Coxiella burnetii*	*IS1111*	–	–	–	–	–	–
*Coxiella*/*Francisella* spp.	*rpoB*	*Coxiella* spp. (98.9–99.2; KP985305) 2 (5N, 1M)	SNC	*Coxiella* spp. (100; KP985329) 16 (24M,38F)	*Coxiella* sp. (95.9; KP985337) 2 (1M, 2F)	*Coxiella* sp. (99.1; KP985331) 1 (1F)	*Coxiella* sp. (97.8;KP985337) 1 (1M)
	*gro*EL	*Coxiella* spp. (99.5; KP985486) 2 (5N, 1M)	*Francisella* sp. (96.8;CP013022, CP012505)^e^ 1 (4F)	*Coxiella* spp. (100; KP985510) 16 (24M, 38H)	*Coxiella* sp. (97.3; KY678195) 2 (1M, 2F)	*Coxiella* sp. (98.2; KY678195) 1 (1F)	*Coxiella* sp. (98.3;CP011126) 1 (1M)
	16S rRNA^c^	NP	*Francisella* sp. (99.6; AB001522) 1 (4F)^f^	*Coxiella* sp. (99.4; JQ480818) 1 (5H)^d^	NP	NP	NP
*Spiroplasma* spp.	*rpoB*	–	–	*Spiroplasma* spp. (99.4; KP967687)^g^ 3 (24M)	–	–	–
	16S rRNA	NP	NP	*Spiroplasma* spp. (98.7–100; KP967685)^h^ 3 (24M)	NP	NP	NP
*Theileria* spp.*/Babesia* spp.	18S rRNA	*Babesia* spp. (91.4; AB734390) 2 (5N, 1M)	–	*B. bigemina* (100; KF606863) 2 (10F)	–	–	–
*Babesia* spp.	ITS 1	*Babesia* spp. (70.9; LK391709) 2 (5N, 1M)	NP	*B. bigemina* (98.8–100; EF458251)^h^ 2 (10H)	NP	NP	NP
	ITS 2	*Babesia* spp. (74.7; EF186914) 2 (5N, 1M)	NP	*B. bigemina* (99.5; EF458266) 2 (10F)	NP	NP	NP

Data show the species names and the highest identity with public sequences (%; GenBank accession number) followed by the number of pools in which the microorganisms have been detected and, in brackets, the number of ticks from each pool

N: nymphs; M: males; F: females; SNC: sequences not conclusive; NP: not performed

^a^Numbers in brackets indicate (number of pools: number of ticks and developmental stage); ^b^two genetic variants were identified; ^c^pan-bacterial PCR assay; ^d^PCR assay performed to four samples, but because this is a pan-bacterial PCR assay (Additional file 1:Table S1), the bacterium was only amplified from one sample; ^e^with 87.6% and 65% query cover, this genotype reached 98.2% and 98.7% identity with *Francisella* sp. detected in soft and hard ticks, respectively (MW287617 and KY678032); ^f^with 92% query cover, this genotype reached 99.8% identity with *Francisella* sp. amplified from *Hyalomma truncatum* (JF290387); ^g^with 42% query cover, the sequences are identical to available *Spiroplasma* sequences from *Rhipicephalus decoloratus* (MK267083-4), but also to those detected in other *Rhipicephalus* and *Ixodes* species (MK267073-7, MK267082, MK267085); ^h^Nucleotide sequences show several ambiguous bases

Twenty-five pools (two *A. variegatum*, three *H. truncatum*, 16 *R. decoloratus*, two *R. duttoni*, one *R. evertsi mimeticus* and one *Rhipicephalus* sp.) were screened for microorganisms.

**Fig. 1:**
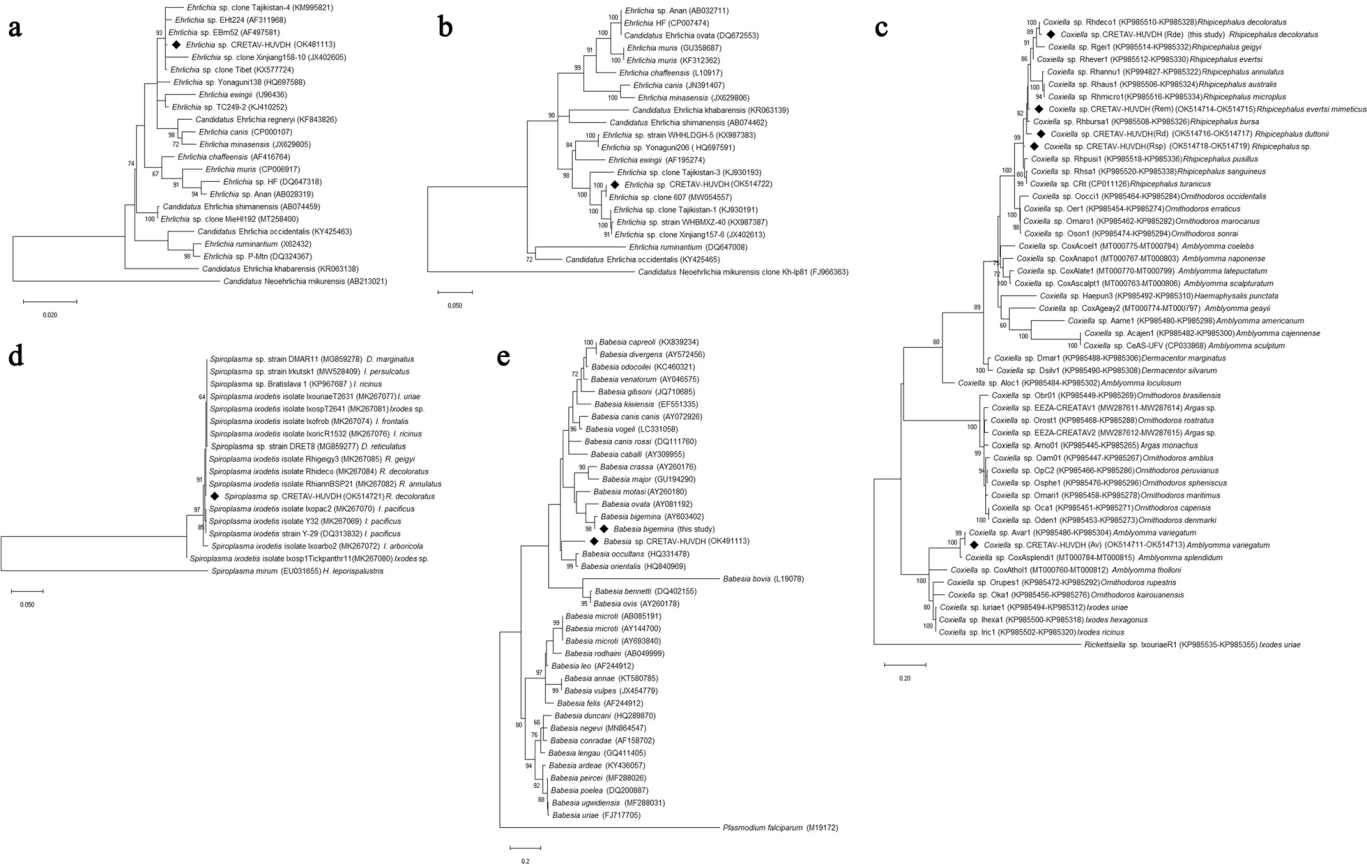
Phylogenetic analysis of the microorganisms detected in this study from ticks collected from cattle in Angola (marked with diamonds). The maximum likelihood trees were obtained using the general time reversible model, a discrete gamma distribution and a proportion of invariable sites (GTR + G + I), with nucleotide substitution selected according to the Akaike information criterion implemented in Mega X. The trees are drawn to scale, with branch lengths measured in the number of substitutions per site. Numbers (> 60%) shown at the nodes correspond to bootstrapped percentages (for 500 repetitions). The GenBank accession numbers of the sequences used in these analyses are shown in brackets. **a** *Ehrlichia* phylogeny was based on 23 partial 16S rRNA gene sequences with a total of 1373 positions in the final dataset. *Candidatus* Neoehrlichia mikurensis was used as an outgroup. **b** *Ehrlichia* phylogeny was based on 22 partial *gro*ESL gene sequences with a total of 1232 positions in the final dataset. *Candidatus* Neoehrlichia mikurensis was used as an outgroup. **c** *Coxiella*-like phylogeny was based on 51 partial *rpoB* and *gro*EL concatenated sequences with a total of 1055 positions in the final dataset. *Rickettsiella* sp. was used as an outgroup. **d** Phylogeny of *Spiroplasma* spp. found in ticks based on 18 partial *rpoB* sequences with a total of 588 positions in the final dataset. **e** Phylogeny of *Babesia* species based on 18S rRNA analysis. The analysis involved 40 nucleotide sequences and a total of 481 positions in the final dataset. *Plasmodium falciparum* was used as outgroup

*Rickettsia* spp. was found in 6/25 pools. According to *ompA*, *Rickettsia africae* was detected in two *A. variegatum* pools and one *R. decoloratus* pool, and *R. aeschlimannii* in three *H. truncatum* pools (Table 2). *Ehrlichia* spp. was found in 6/25 pools of female *R. decoloratus*. Analysis of *gro*ESL, *gltA* and 16S rRNA amplicons revealed the highest identities with unclassified *Ehrlichia* (Table 2, Fig. 1a, b) and showed less than 93.5%, 87.6% and 99.2% identity, respectively, with validated species. Other Anaplasmataceae, *Borrelia* spp. (relapsing fever or Lyme groups) or *Coxiella burnetii* were not detected. Nevertheless, *Coxiella* spp. were found in all but *H. truncatum* pools. For *H. truncatum*, *rpoB* sequences showed inconclusive data, whereas *gro*EL and universal 16S rRNA sequences showed the highest similarity (< 97% and 99.6%, respectively) with *Francisella* sp. in one pool. This 16S rRNA amplicon showed 99.8% identity (92% query cover) with a *Francisella* endosymbiont of *H. truncatum* JF290387 (Table 2). For the remaining tick species, different *Coxiella* genotypes were found. All but two were identical or closely related to public sequences. Genotypes detected in *R. duttoni* and *Rhipicephalus* sp. did not reach > 98.3% identity with *Coxiella* (Table 2, Fig. 1c). *Spiroplasma* sp. was amplified from three *R. decoloratus* male pools (Table 2). According to *rpoB*, this genotype was closely related to *Spiroplasma ixodetis* and related strains of hard ticks (Fig. 1d).

*Babesia bigemina* was identified in two *R. decoloratus* female pools, and *Babesia* sp. was detected in two *A. variegatum* pools, according to 18S rRNA, ITS-1 and ITS-2 analysis (Table 2, Fig. 1e).

## Discussion

This study reports the detection of well-known pathogens *R. africae*, *R. aeschlimannii* and *B. bigemina*, and scarce characterized *Ehrlichia*, *Coxiella*, *Francisella*, *Spiroplasma* and *Babesia* species with unknown pathogenicity in ticks from cattle in Angola.

Our results confirm the circulation of *R. africae* and demonstrate the circulation of *R. aeschlimannii* in Angola. Although *R. aeschlimannii* human infection had been reported from South Africa and *H. truncatum* had been suggested as a vector [16, 17], this pathogen had not been previously found in Angola. African tick-bite fever (ATBF) is endemic in sub-Saharan Africa, but no cases from Angola have been reported [5, 6]. The agent, *R. africae*, has been recently reported from *A. variegatum* in this area [18]. This study confirms the circulation of *R. africae* in the recognized vector, suggesting that ATBF cases could be unreported or misdiagnosed. The presence of *R. africae* in *R. decoloratus* is known, but their role as a vector should be investigated [6, 19]. Moreover, our finding in fed ticks could be due to blood meal or co-feeding.

Only six *Ehrlichia* species are currently recognized, and all but one are responsible for human and/or animal ehrlichiosis [20]. Human ehrlichiosis has been reported from southern Africa, where heartwater (a disease of domestic and wild ruminants caused by *E. ruminantium*) is endemic [6, 8]. Moreover, ‘*Candidatus*’ have been proposed, and *Ehrlichia* genotypes have been partially characterized. Further studies are needed to determine their taxonomic status and pathogenic potential. Herein, a novel *Ehrlichia* genotype has been detected in six *R. decoloratus* pools.

Tick diet based on blood is unbalanced, and endosymbionts (e.g. *Coxiella*-like, *Francisella*-like) provide essential nutrients for ticks [21]. Although virulence genes identified in their pathogenic related species, *C. burnetii* and *Francisella tularensis*, could be absent or non-functional in symbionts, *Coxiella*-like has been considered a pathogen [21–23]. Herein, *Coxiella*-like was detected in all but *H. truncatum* pools, and potential novel *Coxiella* genotypes were detected in *R. duttoni* and *Rhipicephalus* sp. The remaining amplicons showed sequences identical or closely related to *Coxiella-*like previously amplified in the same tick species. *Francisella* sp. was detected in 1/3 *H. truncatum* pools. The sequence was genetically related to a *Francisella* sp. endosymbiont amplicon previously detected in this tick species.

*Spiroplasma* spp. have been found in several hard tick species, and the role of this genus as pathogen has been suggested [24]. Herein, *Spiroplasma* sp. closely related to *S. ixodetis* was detected in 3/16 *R. decoloratus* pools. *Spiroplasma* sp. was previously detected in this species according to a short *rpoB* sequence (Table 2), and this study provides a wider genetic identification.

*Babesia bigemina*, responsible for babesiosis, is prevalent in Angolan cattle [25]. Our study demonstrates its presence in *R. decoloratus* (competent vector) in Angola. Moreover, a potential novel *Babesia* species is circulating in Angolan *A. variegatum*.

*Ehrlichia ruminantium, Anaplasma* and *Theileria* spp. have been reported from Angolan cattle; the latter has been also detected in one *A. variegatum* specimen [8, 18, 25, 26]. Herein, the failure to detect these expected tick-borne microorganisms could be due to the low number of ticks and species analysed, the short period of time for tick collection and/or the host origin.

## Conclusions

This study highlights the importance of ticks in public health in the studied area, and these results should be considered in developing protocols for the management of patients with FUO and for veterinary practices in Angola. Nevertheless, this is only the tip of the iceberg, and more ticks belonging to more species, hosts, from wider areas, etc., as well as broader screening of microorganisms, including viruses (not analysed in this study due to the sample preservation method available), are required to evaluate the risk of tick-borne diseases in Angola.

## Supplementary Information

**Additional file 1: Table S1.** PCR primer pairs and conditions used in this study.

## Data Availability

Novel sequences of this study were deposited on GenBank under accession numbers OK481091-OK481100, OK481107-OK481113, OK491113-OK491116, OK482869-OK482874 and OK514711-OK514725.

## Abbreviations

ATBF: African tick-bite fever
BLAST: Basic Local Alignment Search Tool
COI: Cytochrome oxidase subunit 1
COVID-19: Coronavirus disease 2019
DNA: Deoxyribonucleic acid
FUO: Fever of unknown origin
GenBank: NIH (National Institutes of Health) genetic sequence database
*gltA*: Citrate synthase
*gro*EL: Chaperonin groEL
*gro*ESL: Chaperonin GroEL/GroES complex
ITS: Internal transcribed spacer
NCBI: National Center for Biotechnology Information
*ompA*: Outer membrane protein A
PCR: Polymerase chain reaction
*rpoB*: RNA polymerase beta subunit
rRNA: Ribosomal ribonucleic acid
sp./spp.: Species
