# Supplementary material for: Old zoonotic agents and novel variants of tick-borne microorganisms from Benguela (Angola), July 2017
Source: Parasit Vectors. 2022 Apr 21;15:140. doi: 10.1186/s13071-022-05238-2 (PMC9022410; doi:10.1186/s13071-022-05238-2)
Supplement: Supplementary file 1 — Additional file 1: Table S1. PCR primer pairs and conditions used in this study. [file 13071_2022_5238_MOESM1_ESM.docx]

**Table S1:** PCR primer pairs and conditions used in this study.

| **Organisms** | **Target gene** | **Primer sequence (5’🡪 3’)** | **Fragment**  **size (bp)** | **Tm (ºC)** | **Reference** |
| --- | --- | --- | --- | --- | --- |
| **Ticks** | 16S rRNA | F:CTGCTCAATGATTTTTTAAATTGCTGTGG  R:CCGGTCTGAACTCAGATCAAGT | 456 | 48  54 | [1] |
|  | 12S rRNA^†^ | F:AAACTAGGATTAGATACCCT  R:AATGAGAGCGACGGGCGATGT | 338 | 51  53 | [2] |
|  | COI^†^ | F:GGTCAACAAATCATAAAGATATTGG  R:TAAACTTCAGGGTGACCAAAAAATCA | 710 | 40 | [3] |
| **Spotted Fever group *Rickettsia* spp.** | *ompA* | F:ATGGCGAATATTTCTCCAAAA  R:GTTCCGTTAATGGCAGCATCT | 631 | 46 | [4,5] |
|  |  | F:ATGGCGAATATTTCTCCAAAA  R:AGTGCAGCATTCGCTCCCCCT | 532 | 48 |  |
| ***Anaplasma/Ehrlichia*/ *Neoehrlichia* spp.** | *gro*ESL | F:AITGGGCTGGTAITGAAAT  R:CCICCIGGIACIAIACCTTC | 1,350 | 48 | [6] |
|  |  | F:ATWGCWAARGAAGCATAGTC  R:CTCAACAGCAGCTCTAGTAGC | 1297 | 75 |  |
| ***Ehrlichia* spp.** | *gltA* | F:ATGTCTACTGCTGCTTGTGA  R:ATGACCAGTATATAACTGACGTG | 1098 | 50 | [7,8] |
|  |  | F:ATGTCTACTGCTGCTTGTGA  R: GAGCAGACCAACCAGATGTTC | 1030 | 50 |  |
|  | 16S rRNA | F:GAACGAACGCTGGCGGCAAGC  R:AGTAYCGRACCAGATAGCCGC | 696 | 50 | [8,9] |
| ***Borrelia burgdorferi* s.l. (Lyme group)** | *flaB* | F :AARGAATTGGCAGTTCAATC  R :GCATTTTCWATTTTAGCAAGTGATG | 497 | 52 | [10,11] |
|  |  | F :ACATATTCAGATGCAGACAGAGGTTCTA  R :GAAGGTGCTGTAGCAGGTGCTGGCTGT | 398 | 55 |  |
| **Relapsing fever group *Borrelia* spp.** | *glpQ* | F:ATGGGTTCAAACAAAAAGTCACC  R:CATTACTGTGTCAGTAAAATCTGTAAATATACCATCTAC | 920 | 70 Touchdown  60 | [12] |
|  |  | F:ATGGGTTCAAACAAAAAGTCACC  R:CCAGGGTCCAATTCCATCAGAATATTGTGCAAC | 700 | 53 | [13] |
| ***Coxiella burnetii*** | *IS1111* | F:TATGTATCCACCGTAGCCAGTC  R:CCCAACAACACCTCCTTATTC | 685 | 48 | [14] |
| ***Coxiella*/*Rickettsiella* spp.** | *rpoB* | F:GGGCGNCAYGGWAAYAAAGGSGT  R:CACCRAAHCGTTGACCRCCAAATTG | 619 | 56 | [15] |
|  |  | F:TCGAAGAYATGCCYTATTTAGAAG  R:AGCTTTMCCACCSARGGGTTGCTG | 542 | 56 |  |
|  | *groEL*^†^ | F:TTTGAAAAYATGGGCGCKCAAATGGT  R:CGRTCRCCAAARCCAGGTGC | 655 | 56 | [15] |
|  |  | F:GAAGTGGCTTCGCRTACWTCAGACG  R:CCAAARCCAGGTGCTTTYAC | 619 | 56 |  |
| ***Spiroplasma* spp.** | *rpoB* | F:GGNTTTATTGAAACACCATAYCGTC  R:GCATGTAATTTATCATCAACCATGTGTG | 1443 | 63 Touchdown | [16] |
|  | 16S rRNA^†^ | F:AGAGTTTGATCCTGGCTCAG  R:TAGCCGTGGCTTTCTGGTAA | ˜500 | 55 | [17] |
| ***Babesia*/*Theileria* spp.** | 18S rRNA | F:GACACAGGGAGGTAGTGACAAG  R:CTAAGAATTTCACCTCTGACAGT | 400 | 51 | [18] |
|  | ITS1^†^ | F:CGAGTGATCCGGTGAATTATTC  R:CCTTCATCGTTGTGTGAGCC | 615 | 52 | [19,20] |
|  | ITS2^†^ | F:GGCTCACACAACGATGAAGG  R:CTCGCCGTTACTAAGGGAATC | 315 | 54 | [19,20] |
| **Pan-bacterial**^†^ | 16S rRNA | F:AGAGTTTGATCCTGGCTCAG  R:ACGGCTACCTTGTTACGACTT | 1500 | 60 | [21] |
| F: Forward; R: Reverse; bp: base pairs; Tm: melting temperature; W= A/T; R= G/A; Y= C/T; N= A/C/G/T; S= G/C; H=A/C/T; M= K/C; K= G/T.  ^†^Performed only with samples with inconclusive results for further characterization. | | | | | |

# References

1. Black WC, Piesman J. Phylogeny of hard and soft tick taxa (Acari:Ixodida) based on mitochondrial 16S rDNA sequences. Proc. Natl. Acad. Sci. USA. 1994; 91:10034-48. doi: 10.1073/pnas.91.21.10034
2. Beati L, Keirans JE. Analysis of the systematic relationships among ticks of the genera *Rhipicephalus* and *Boophilus* (Acari: Ixodidae) based on mitochondrial 12S ribosomal DNA gene sequences and morphological characters. J Parasitol. 2001; 87:32-48. doi: 10.1645/0022-3395(2001)087[0032:AOTSRA]2.0.CO;2
3. Folmer O, Black M, Hoeh W, Lutz R, Vrijenhoek R. DNA primers for amplification of mitochondrial cytochrome c oxidase subunit I from diverse metazoan invertebrates. Mol Mar Biol Biotechnol. 1994;3:294-9.
4. Roux V, Fournier PE, Raoult D. Diffentiation of Spotted Fever Group Rickettsiae by Sequencing and Analysis of Restriction Fragment Length Polymorphism of PCR-Amplified DNA of the Gene Encoding the Protein rOmpA. J Clin Microbiol. 1996;34:2058-65. doi: 10.1128/jcm.34.9.2058-2065.1996.
5. Regnery RL, Spruill CL, Plikaytis BD. Genotypic identification of rickettsiae and estimation of intraspecies sequence divergence for portions of two rickettsial genes. J Bacteriol. 1991; 173:1576-89. Doi: 10.1128/jb.173.5.1576-1589.1991
6. Liz JS, Sumner JW, Pfister K, Brossard M. PCR detection and serological evidence of granulocytic ehrlichial infection in roe deer (*Capreolus capreolus)* and chamois (*Rupicapra rupicapra*). J Clin Microbiol. 2002; 40:892–7. doi: 10.1128/JCM.40.3.892-897.2002
7. Cabezas Cruz AC, Zweygarth E, Ribeiro MF, da Silveira JA, de la Fuente J, Grubhoffer L, et al. New species of *Ehrlichia* isolated from *Rhipicephalus* (*Boophilus*) *microplus* shows an ortholog of the *E. canis* major immunogenic glycoprotein gp36 with a new sequence of tandem repeats. Parasit Vectors. 2012;5:291. doi: 10.1186/1756-3305-5-291
8. Rar VA, Pukhovskaya NM, Ryabchikova EI, Vysochina NP, Bakhmetyeva SV, Zdanovskaia NI, et al. Molecular-genetic and ultrastructural characteristics of *'Candidatus* Ehrlichia khabarensis', a new member of the *Ehrlichia* genus. Ticks Tick Borne Dis. 2015; 6:658-67. doi: 10.1016/j.ttbdis.2015.05.012.
9. Rar VA, Livanova NN, Panov VV, Doroschenko EK, Pukhovskaya NM, Vysochina NP, et al. Genetic diversity of *Anaplasma* and *Ehrlichia* in the Asian part of Russia. Ticks Tick Borne Dis. 2010;1:57-65. doi: 10.1016/j.ttbdis.2010.01.002.
10. Clark K, Hendricks A, Burge D. Molecular Identification and Analysis of *Borrelia burgdorferi* sensu lato in lizards in the Southeastern United States. Applied and Environmental Microbiol. 2005; 71:2616-25. doi. 10.1128/AEM.71.5.2616-2625.2005
11. Johnson BJB, Happ CM, Mayer LW, Piesman J. Detection of *Borrelia burgdorferi* in ticks by species-specific amplification of the flagellin gene. Am J Trop Med Hyg. 1992; 47:730-41. doi: 10.4269/ajtmh.1992.47.730.
12. Hovius JW, de Wever B, Sohne M, Brouwer MC, Coumou J, Wagemakers A, et al. A case of meningoencephalitis by the relapsing fever spirochaete *Borrelia miyamotoi* in Europe. Lancet. 2013;382:658. doi: 10.1016/S0140-6736(13)61644-X.
13. Wagemakers A, Jahfari S, de Wever B, Spanjaard L, Starink MV, de Vries HJ, et al. *Borrelia miyamotoi* in vectors and hosts in The Netherlands. Ticks Tick Borne Dis. 2017:8:370-4. doi: 10.1016/j.ttbdis.2016.12.012.
14. Massung R, Slater K, Owens JH, Nicholson WL, Mather TN, Solberg VB, et al. Nested PCR assay for detection of granulocytic ehrlichiae. J Clin Microbiol. 1998;36:1090-95. Doi: 10.1128/JCM.36.4.1090-1095.1998
15. Duron O, Noël V, McCoy KD, Bonazzi M, Sidi-Boumedine K, Morel O, et al. The Recent Evolution of a Maternally-Inherited Endosymbiont of Ticks Led to the Emergence of the Q Fever Pathogen, *Coxiella burnetii*. PLoS Pathog. 2015;11:e1004892. doi: 10.1371/journal.ppat.1004892.
16. Haselkorn TS, Markow TA, Moran NA. Multiple introductions of the *Spiroplasma* bacterial endosymbiont into *Drosophila*. Mol Ecol. 2009;18:1294–305. Doi: 10.1111/j.1365-294X.2009.04085.x
17. Fukatsu T, Nikoh N. Endosymbiotic microbiota of the bamboo pseudococcid *Antonina crawii* (Insecta, Homoptera) Appl. Environ Microbiol. 2000;66:643–50. doi: 10.1128/AEM.66.2.643-650.2000
18. Gubbels JM, de Vos AP, van der Weide M, Viseras J, Schouls LM, de Vries E, Jongejan F. Simultaneous detection of bovine *Theileria* and *Babesia* species by reverse line blot hybridization. J Clin Microbiol. 1999;37:1782-9. doi: 10.1128/JCM.37.6.1782-1789.1999
19. Blaschitz M, Narodoslavsky-Gföller M, Kanzler M, Stanek G, Walochnik J. *Babesia* species occurring in Austrian *Ixodes ricinus* ticks. Appl Environ Microbiol. 2008;74:4841-4. doi: 10.1128/AEM.00035-08.
20. Bajer A, Alsarraf M, Bednarska M, Mohallal EM, Mierzejewska EJ, Behnke-Borowczyk J, et al. *Babesia behnkei* sp. nov., a novel *Babesia* species infecting isolated populations of Wagner's gerbil, *Dipodillus dasyurus*, from the Sinai Mountains, Egypt Parasit Vectors. 2014;7:572. doi: 10.1186/s13071-014-0572-9.
21. Weisburg WG, Barns SM, Pelletier DA, Lane DJ. 16S ribosomal DNA amplification for phylogenetic study. J Bacteriol. 1994;173:697–703. doi: 10.1128/jb.173.2.697-703.1991.
